# The gp44 Ejection Protein of *Staphylococcus aureus* Bacteriophage 80α Binds to the Ends of the Genome and Protects It from Degradation

**DOI:** 10.3390/v12050563

**Published:** 2020-05-20

**Authors:** Keith A Manning, Terje Dokland

**Affiliations:** Department of Microbiology, University of Alabama at Birmingham, Birmingham, AL 35294, USA; k1797732@uab.edu

**Keywords:** minor capsid protein, pilot protein, genome replication, recombinase, exonuclease, lytic–lysogenic switch

## Abstract

Bacteriophage 80α is a representative of a class of temperate phages that infect *Staphylococcus aureus* and other Gram-positive bacteria. Many of these phages carry genes encoding toxins and other virulence factors. This phage, 80α, is also involved in high-frequency mobilization of *S. aureus* pathogenicity islands (SaPIs), mobile genetic elements that carry virulence factor genes. Bacteriophage 80α encodes a minor capsid protein, gp44, between the genes for the portal protein and major capsid protein. Gp44 is essential for a productive infection by 80α but not for transduction of SaPIs or plasmids. We previously demonstrated that gp44 is an ejection protein that acts to promote progression to the lytic cycle upon infection and suggested that the protein might act as an anti-repressor of CI in the lytic–lysogenic switch. However, an 80α Δ44 mutant also exhibited a reduced rate of lysogeny. Here, we show that gp44 is a non-specific DNA binding protein with affinity for the blunt ends of linear DNA. Our data suggest a model in which gp44 promotes circularization of the genome after injection into the host cell, a key initial step both for lytic growth and for the establishment of lysogeny.

## 1. Introduction

*Staphylococcus aureus* is a Gram-positive bacterium and an opportunistic human pathogen responsible for a broad range of diseases [1]. The emergence of antibiotic resistance in *S. aureus* and other bacteria, combined with the lack of new antibiotics in the development pipeline, has become a major public health concern [2]. This has led to a renewed interest in phage therapy as an alternative to antibiotic treatment. However, bacteriophages are also the main mediators of gene transfer and horizontal evolution in *S. aureus* and are often associated with virulence factors [3,4].

Bacteriophage 80α (GenBank ID: DQ517338) is a representative of a class of temperate phages that infect *S. aureus* and other Gram-positive bacteria [5]. Many phages in this group carry genes encoding superantigen toxins and other virulence factors [3,6,7]. 80α is also involved in high-frequency mobilization of *S. aureus* pathogenicity islands (SaPIs), mobile genetic elements that carry genes encoding virulence factors [8].

In its integrated (prophage) state, 80α is repressed through the expression of its immunity repressor, CI, which binds to an operator in the *cI*–*cro* intergenic region of the lytic–lysogenic switch [5,9,10]. Induction of the phage via the SOS response leads to cleavage of CI and initiation of the lytic program. During an 80α infection, the lytic cycle is strongly favored, suggesting that CI levels are normally too low to establish lysogeny [11].

Upon induction of lytic growth, empty precursor procapsids are assembled from a major capsid protein (CP, gp47) and a portal protein (PP, gp42), together with a scaffolding protein (SP, gp46) that acts as a chaperone in the assembly process [12,13]. Phage DNA is packaged into these procapsids by a headful mechanism that utilizes the terminase complex, consisting of small (TerS, gp40) and large (TerL, gp41) subunits [14]. The substrate for the packaging is a linear concatemer of phage genomes. TerS recognizes a specific *pac* site in the genome [15] to initiate the packaging, while TerL carries out the packaging itself. The packaging proceeds until the capsid is full, upon which the nuclease activity of TerL cleaves the DNA, presumably in a manner similar to phage P22 [16]. Each capsid is typically filled with ≈110% of a genome, leading to circularly permuted, terminally redundant DNA in the virion. Such redundancy is essential for circularization of the genome by recombinases after injection into the host [17,18]. Circularization is required both for genome replication and progression toward lytic growth and for integration and establishment of lysogeny.

80α and many other phages of the Firmicutes encode a minor capsid protein between the genes encoding PP and SP [11,19]. We previously showed that this protein, encoded by 80α open reading frame 44 (ORF44) and thus referred to as gp44, is incorporated into procapsids at low copy numbers (≈2 copies/capsid) and is vital to successful replication of the virus post-infection [11]. However, gp44 is not necessary for the transduction of plasmids or SaPIs. We showed that a mutant phage with a deletion of ORF44 (80α Δ44) assembled and packaged phage DNA normally and was able to transfer the phage genome to a naïve cell but did not enter productive infection at a normal rate [11]. However, lytic growth could be recovered if the cells were also induced with mitomycin C, which normally causes SOS induction and depression of the CI immunity repressor. Based on these results, we hypothesized that gp44 was involved in the lytic/lysogenic decision, either by interfering with CI binding to its operator sequence, or by inhibiting production of the CI protein [11]. However, we also observed that the 80α Δ44 mutant was *less* likely to undergo lysogeny than the wildtype phage, suggesting that the effect of gp44 was more indirect, for example by promoting survival of the phage DNA after injection.

Here, we have further characterized the gp44 protein in terms of its domain structure, oligomerization, CI interaction, and DNA binding properties. We found that, contrary to our original hypotheses, purified gp44 did not bind directly to CI and did not bind specifically to the *cI*–*cro* intergenic region. Instead, gp44 bound to the blunt ends of linear DNA in a non-sequence specific manner and could protect the DNA from cleavage by exonuclease. Our data are consistent with a model in which gp44 promotes circularization of the genome after injection into the host, a necessary initial step both for a productive lytic infection and for the establishment of lysogeny.

## 2. Materials and Methods

### 2.1. Cloning, Expression, and Purification of gp44 and CI

Relevant gene fragments were amplified by PCR from 80α genomic DNA and inserted into the pET21a vector or the pET21a-based pE-SUMO vector (Table 1) for expression in *Escherichia coli* strain BL21(DE3). The cells harboring the expression plasmids were grown at 37 °C until A_600_ = 0.6, induced by the addition of IPTG to 1 mM and grown for an additional 2–3 hours. Cells were harvested by centrifugation at 7000× *g* for 10 min at 4 °C and the resulting pellet was kept frozen at −20 °C overnight. The cells were resuspended on ice in lysis buffer (100 mM Na phosphate pH 7.6, 500 mM NaCl, 5 mM MgCl_2_, and 10% glycerol) with 10 mM imidazole and 60 units/mL Benzonase^TM^ nuclease (MilliporeSigma, St. Louis, MO, USA), and lysed with an Avestin EmulsiFlex B-15 high-pressure cell disruptor with a minimum of three passages. Precipitated protein and unlysed cells were removed by centrifugation at 17,000× *g* for 30 min at 4 °C. The clarified lysates (25 mL) were added to 5 mL Ni–NTA resin, washed with four volumes of lysis buffer with 10 mM imidazole, and eluted with steps of 50–300 mM imidazole. Untagged proteins were produced from the His–SUMO fusion proteins by cleavage with 0.1 mg/mL Ulp1 for 3 h at 30 °C, followed by removal of the His–SUMO moiety on an Ni–NTA column. For binding experiments, untagged and tagged proteins were incubated for 30 min at 22 °C and run over an Ni–NTA column or analyzed by size exclusion chromatography (SEC). For SEC separation, the affinity purified protein was loaded on either a Superdex 75 Increase 10/300 GL or a Superdex 200 10/300 GL column (GE Research, Niskayuna, NY, USA), using a Bio-Rad DuoFlow system.

### 2.2. Complementation Analysis

For complementation in *S. aureus*, the various gene fragments were inserted into the IPTG-inducible vector pG164 and introduced into *S. aureus* strain Sa178RI, as previously described [11]. The transformed cells were plated on TSA with 25 µg/mL chloramphenicol. Aliquots (10 µL) from a dilution series of CsCl-purified 80α WT or 80α Δ44 phage produced from the lysogens RN10616 or ST64, respectively, were added to plates containing the transformed cells in the presence or absence of 1 mM IPTG [11]. Plaques were counted after 18–20 h of growth at 32 °C.

### 2.3. Mass Spectrometry Analysis

For full-length mass determination by ESI-MS, the SEC-purified proteins were loaded on a C8 reverse phase column after adding formic acid to 0.1% final concentration and eluted with a gradient of acetonitrile into a Waters Synapt mass spectrometer. For protein identification from gel bands, the SEC-purified gp44 sample was separated by electrophoresis on a 10% Bis–Tris gel. The bands were excised, digested with trypsin, and subjected to LC-MS analysis, using a Thermo Finnigan LTQ XL spectrometer and a Mascot search of the UniRef100 database. Only spectra with a >80% peptide identification probability as determined by Scaffold Viewer 3 were counted for sequence coverage.

### 2.4. DNA Binding and Protection Assays

DNA fragments corresponding to the 163 base pair *cI–cro* intergenic region (IGR DNA), a 1136 base pair region including the entire *cI* through *cro* genes (*cI–cro* DNA), and a 1018 base pair sequence derived from the *fibU* (ORF68) tail fiber gene (TF DNA) were produced by PCR from 80α genomic DNA using DreamTaq polymerase (Thermo Fisher, Waltham, MA, USA) and purified by QIAQuick Nucleotide Removal kit (Qiagen, Hilden, Germany). These DNAs, at 21–25 nM concentration, were incubated with up to 1217-fold molar excess of either gp44 or CI protein in an electrophoretic mobility shift assay (EMSA) buffer (10 mM Na phosphate pH 7.6, 100 mM NaCl, 1 mM MgCl_2_, and 5% glycerol) with 0.5 mg/mL BSA for 15 min at 22 °C. The mixture was then run on either a Novex 6% polyacrylamide DNA retardation gel (Thermo Fisher) or on 0.7% agarose gel in 0.5× or 1× TBE buffer, respectively, and stained with ethidium bromide for DNA detection. For nuclease assays, the DNA–protein mixture was incubated with 0.025 U T5 exonuclease or 0.5 µg DNAse I and incubated for 2 hours at 30 °C. The reaction was halted by addition of EDTA (final concentration 14 mM) and proteinase K (final concentration 0.3 mg/mL) and incubated at 55 °C for 1 hour. The resulting mixture was then run on 0.7% agarose gels and stained with ethidium bromide for DNA detection. The 80α genomic DNA was incubated with gp44 or CI, cleaved with 0.8 U EcoRI (New England Biolabs, Ipswich, MA, USA) or 1 µL FastDigest EcoRV (Thermo Scientific) for 60 min at 30 °C, and subjected to the same assay.

## 3. Results

### 3.1. Structure Prediction of gp44

**Figure 1:**
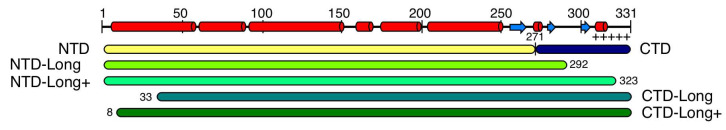
Domain structure of 80α gp44. α-helices and β-strands predicted by I-TASSER are shown as cylinders and arrows, respectively. The cluster of positively charged residues near the C-terminus is indicated (+++). The bars labeled NTD and CTD correspond to the two parts that were found in the purified gp44 sample by MS. The remaining bars show the various truncated constructs that were generated.

Homologs of gp44 are widespread among the *Caudovirales*, but appear to be specific to headful packaging phages that infect Gram-positive Firmicutes [11]. However, no homologs of gp44 could be identified in the protein structure databank (PDB) by HHpred [20]. Low-confidence hits (E-value > 10) identified by HHpred in the N-terminal region of gp44 included mainly α-helical coiled-coil proteins. De novo structure predictions with I-TASSER [21], Phyre2 [22], RaptorX [23], and IntFOLD [24] gave greatly differing, low-confidence results (Figure S1). Nevertheless, there were commonalities between the predictions: All programs predicted a predominantly α-helical N-terminal region from residue 1 to about 250, followed by a mostly unstructured C-terminal region that often included a few short α-helices and β-strands (Figure 1). The protein includes a large number of positively charged residues, with lysines and arginines constituting 17.5% of the amino acid composition. Positively charged residues are more concentrated toward the C-terminal end, comprising a third of the last 30 residues of the protein.

### 3.2. Gp44 Has a Two-Domain Structure and Forms a Dimer in Solution

**Figure 2:**
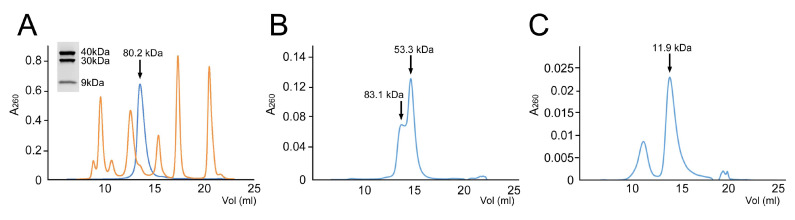
Purification of gp44. (**A**) Size exclusion chromatography (SEC) profile of full-length gp44 (blue) superimposed on a protein standard (orange; peaks at 670, 158, 44, 17, and 1.25 kDa). The gp44 peak corresponds to an apparent MW of 80.2 kDa (arrow), close to the predicted 78.8 kDa for a gp44–His dimer. The insert shows an SDS-PAGE of protein collected from the SEC peak. The three bands corresponding to full-length (FL) gp44 (apparent MW 40 kDa), gp44-NTD (30 kDa) and gp44-CTD (9 kDa) are indicated. (**B**,**C**) SEC profiles of gp44-NTD-His (**B**) and gp44-CTD-His (**C**). The main peaks and their apparent masses are indicated.

The full-length ORF44 gene was cloned into a pET21 expression vector, with a His_6_-tag on the C-terminus, generating plasmid pKM1 (Table 1). After expression, the protein was purified by Ni–NTA affinity. The C-terminally His-tagged gp44, referred to in the following as gp44–His, bound strongly to Ni–NTA resin, eluting at 300 mM imidazole after a 100 mM imidazole wash. This material was further purified by SEC on a Superdex 200 column and eluted as a single peak at 13.6 mL, corresponding to 80.2 kDa, close to the predicted 78.8 kDa mass for a gp44–His dimer (Figure 2A). When the purified protein was analyzed by SDS-PAGE, three bands appeared on the gel: a band with apparent molecular weight of ≈40 kDa, corresponding to the full-length gp44 (predicted mass 39.3 kDa), and two smaller bands at ≈30 and ≈9 kDa, suggesting that the protein had been cleaved into two domains (Figure 2A, Inset).

The SEC-purified material was subjected to electrospray ionization mass spectrometry (ESI-MS) analysis, which showed two major peaks at 39,169 and 31,589 Da, corresponding to the predicted masses of the full-length gp44–His protein and an N-terminal fragment, comprising residues 2–271, and consistent with the apparent masses of the large and middle bands observed by SDS-PAGE. No mass corresponding to the smaller band, assumed to be the C-terminal part, could be detected by ESI-MS, presumably because it did not bind to the reverse phase column used in the ESI-MS experiment. MALDI-MS on tryptic digests of the two bands cut out from the SDS-PAGE gel showed peptides corresponding to the C-terminal region of gp44 predominantly in the small band, and peptides corresponding to the N-terminal region predominantly in the large band. Some overlap is presumably caused by peptides diffusing through the gel (Figure S2).

We do not know why gp44 became cleaved, but it was assumed to be caused by some protease present in the *E. coli* lysate. The cleavage was consistent between different preparations, and we were unable to prevent it by adding protease inhibitors prior to lysis. In light of the sequence analysis, the results suggest that gp44 has two domains separated by a flexible hinge region that is sensitive to cleavage. The observation that the fragments were not separated by SEC shows that the protein is held together by non-covalent interactions and that the dimeric structure is retained after cleavage.

To characterize further the predicted two-domain structure of gp44, we generated expression constructs corresponding to the N-terminal (gp44-NTD-His, residues 1–271) and C-terminal (gp44-CTD-His, 272–331) domains identified by the ESI-MS analysis (Figure 1; Table 1). Both constructs included His_6_-tags at the C-terminus. The proteins were expressed in *E. coli* and purified by Ni–NTA affinity. The gp44-NTD-His protein was run over a Superdex 200 SEC column, where it separated as two overlapping peaks at 13.7 and 14.7 mL, corresponding to 83.1 and 53.3 kDa, respectively (Figure 2B). These peaks most likely correspond to dimers and monomers of gp44-NTD-His, with calculated masses of 65.0 and 32.5 kDa, respectively. The discrepancy between the calculated masses and those observed by SEC is probably due to gp44 having an unusually elongated shape. The gp44-CTD-His protein was run on a Superdex 75 column. Most of the material separated in a peak at 13.8 mL, corresponding to 11.9 kDa (Figure 2C). A smaller peak at 11.0 mL (38.2 kDa) was shown to contain mostly a ≈30 kDa *E. coli* contaminant. The predicted mass of a gp44-CTD-His monomer is 7.7 kDa and a dimer would be 15.4 kDa; these fall on either side of the SEC peak. Most likely, the protein runs aberrantly due to an unusual shape or high degree of disorder, consistent with the sequence analysis (Figure S1).

### 3.3. All of gp44 Is Required for Function In Vivo

We then tested the capabilities of the two presumptive domains in vivo using a trans-complementation assay. In this assay, 80α Δ44 mutant phages are plated on bacteria expressing the full-length or truncated gp44 proteins from pG164-derived plasmids in the presence of IPTG, as previously described [11]. Functional complementation leads to plaque formation. As a control, 80α WT was plated on the gp44 full-length complementing strain (KM12), resulting in a titer of 10^10^ PFU/mL, which is typical for wildtype phage (Table 2). Meanwhile, 80α Δ44 plated on the same strain in the absence of IPTG yielded no titer, but in the presence of IPTG resulted in a titer of 10^8^ PFU/mL, showing that the Δ44 defect had been complemented by the full-length gp44 protein (Table 2). The lack of full complementation to wildtype levels is likely because gp44 needs to be packaged in the virions for full functionality, as we previously showed [11]. In contrast, the N- and C-terminal domains separately were unable to complement 80α Δ44, yielding no titers (Table 2). We then made a series of truncations where fewer residues were removed from the full-length protein, including gp44-NTD-Long (residues 1–292) and gp44-CTD-Long (residues 33–331; Figure 1). These truncations were still unable to produce plaques (Table 2). Indeed, only very small truncations of 7 and 8 amino acids from the N- and C-termini, respectively (gp44-CTD-Long+, residues 8–331; NTD-Long+, residues 1–323), were capable of complementing the 80α Δ44 deletion (Table 2). These results show that although gp44 might have a two-domain organization, neither domain is sufficient, and essentially the whole gp44 protein is required for full function in vivo.

### 3.4. Gp44 Does Not Interact Directly with the CI Repressor

One of the previously proposed models for gp44 activity was that it acted as an anti-repressor through direct interaction with the CI repressor [11]. Like most repressors, CI was expected to form a dimer, as had been demonstrated with the phage Φ11 CI protein, which is 100% identical to 80α CI in the C-terminal 146 residues, but has a divergent N-terminal domain [5]. Binding between gp44 and CI might disrupt both dimers, as we previously showed for the interaction between the SaPI1 Stl repressor and the ΦNM1 anti-repressor Dut [27].

**Figure 3:**
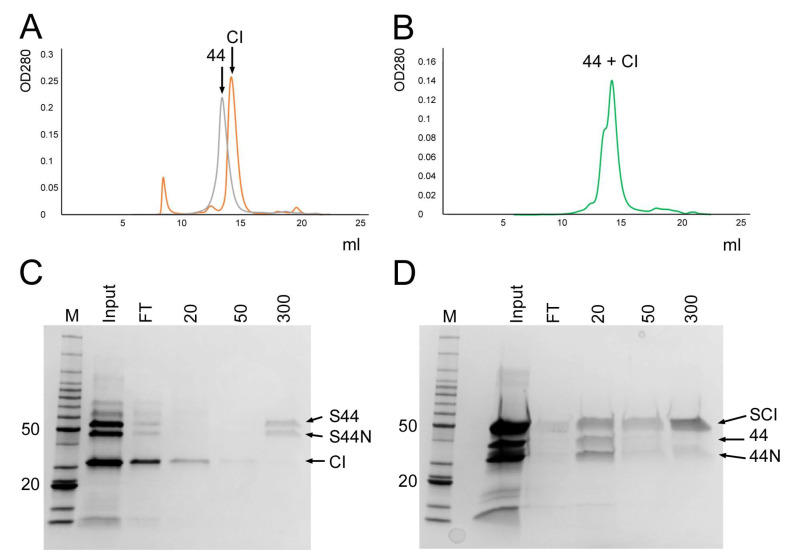
Binding of gp44 to CI. (**A**) Superimposed SEC profiles of the untagged CI (orange) and gp44 (gray) proteins, purified by Ulp1 cleavage from the His-SUMO fusion proteins. (**B**) SEC profile of the CI–gp44 mixture, showing that no new peaks corresponding to a CI–gp44 complex appeared. (**C**,**D**) SDS-PAGE of eluates from affinity pulldowns of untagged CI with His-SUMO-gp44 (**C**), and untagged gp44 with His-SUMO-CI (**D**). M, marker; FT, flow-through. Sequential elutions with 20, 50, and 300 mM imidazole are shown. The bands corresponding to His-SUMO-gp44 (S44), His-SUMO-gp44-NTD (S44N), untagged CI (CI), His-SUMO-CI (SCI), untagged gp44 (44), and untagged gp44-NTD (44N) are indicated.

To test this hypothesis, we first generated a plasmid (pKM22) for expression of a His-SUMO-CI fusion protein (Table 1). After purification by Ni–NTA affinity, the His-SUMO-CI construct allowed the production of untagged CI by cleavage with ubiquitin-like protease Ulp1 followed by removal of the His-SUMO fragment by Ni–NTA affinity. After cleavage, the purified CI protein was separated by SEC on Superdex 200 column, resulting in a peak at 58.7 kDa (Figure 3A), consistent with a CI dimer (predicted mass 53.8 kDa). We also generated a His-SUMO-gp44 construct (pKM23, Table 1) for production of untagged gp44 by the same method, which ran at the expected position for a full-length gp44 dimer by SEC (Figure 3A).

To test for a direct interaction between gp44 and CI, the two untagged proteins were mixed, incubated for 30 min at 22 °C and run over the same Superdex 200 SEC column. We expected that if there was an interaction between the two proteins, it would either be between CI and gp44 monomers, like the Stl–Dut interaction [27], or between dimers. In the first case, we should observe an attenuation of the individual dimer peaks and a rise of a peak in between, corresponding to the predicted 65.4 kDa heterodimer, whereas in the second case, we would observe a new peak at 131 kDa, corresponding to the heterotetramer. However, no shifted or new peaks were detected by SEC analysis (Figure 3B), suggesting that the two proteins did not interact.

We also carried out an affinity pull-down to test for an interaction between gp44 and CI. Firstly, the His-SUMO-gp44 fusion protein was incubated with untagged CI for 30 min at 22 °C and run over an Ni-NTA column. The uncleaved His-SUMO-gp44 was chosen for this experiment rather than gp44–His, to avoid confusion between the gp44-NTD and CI, which have similar mobility by SDS-PAGE—like the full-length gp44, the His-SUMO-gp44 protein gets partially cleaved into an N-terminal fragment (His-SUMO-gp44-NTD) and a gp44 C-terminal fragment during this experiment. The His-SUMO-gp44-NTD is easily distinguishable from CI (Figure 3C). (The very small gp44-CTD fragment is not clearly seen on the gel.) Untagged CI eluted in the flow-through and the 50 mM imidazole wash, whereas His-SUMO-gp44 and His-SUMO-gp44-NTD remained bound to the resin and eluted at 300 mM imidazole, showing that the two proteins did not interact (Figure 3C). In the inverse experiment, untagged gp44 was incubated with His-SUMO-CI fusion protein and loaded on the Ni–NTA column. In this case, gp44 and gp44-NTD eluted in the flow-through, while His-SUMO-CI was retained on the column, and eluted at 300 mM imidazole, again indicating that no interaction between the two proteins occurred (Figure 3D).

From these results, we concluded that our earlier hypothesis of gp44 interference with CI repression through a direct gp44–CI protein interaction was incorrect. Although it is still possible that the two proteins interact with an affinity too low to detect by this approach, this seems unlikely. To function effectively as an anti-repressor able to displace CI from its operator, any presumptive gp44–CI interaction would be expected to be quite strong. In fact, other data (see below) suggested a different mechanism.

### 3.5. Gp44 Binds DNA

An alternative hypothesis that we had previously considered was that gp44 was a DNA binding protein that either blocked CI from binding to its operator sequence or functioned as a repressor of CI expression. Both these possibilities would imply binding of gp44 to an operator sequence in the intergenic region (IGR) between the *cI* and *cro* genes. To test this hypothesis, we used an electrophoretic mobility shift assay (EMSA) to test for binding of gp44 to a 163 base pair DNA that corresponded to the *cI–cro* IGR. As a positive control for binding, we used CI itself, whose homolog in Φ11 had been well characterized and shown to bind to three separate sequences within the IGR [10].

**Figure 4:**
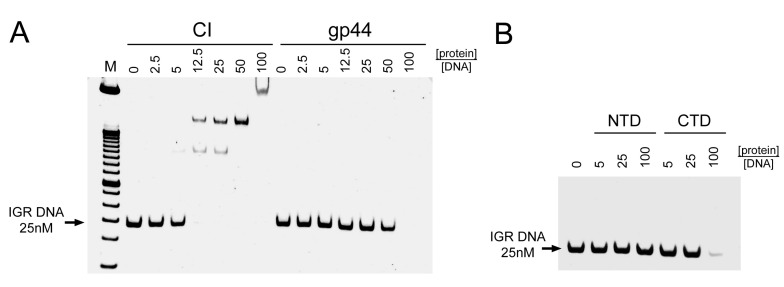
DNA binding of gp44 and CI. (**A**) Separation on a 6% polyacrylamide DNA retardation gel of 25 nM intergenic region (IGR) DNA after incubation with varying concentrations of CI (left) or gp44 (right). (**B**) IGR DNA incubated with gp44-NTD or gp44-CTD. The protein concentrations are given above the gel as the -fold molar excess of protein to DNA. The position of the IGR DNA band is indicated.

The IGR DNA was incubated with varying amounts of purified CI or gp44 and loaded on a Novex DNA retardation 6% polyacrylamide gel. As expected, CI caused a concentration-dependent shift of the IGR DNAs, with a complete shift seen with CI:DNA ratios of ≥12.5:1 (Figure 4A). In contrast, gp44 did not lead to the appearance of a shifted band, although a slight concentration-dependent weakening of the band could be observed. At a gp44:DNA ratio of 100:1, the DNA band disappeared completely (Figure 4A).

We also tested DNA binding of the gp44 N- and C-terminal domains separately. Incubation with gp44-NTD-His had no effect on the electrophoretic mobility of the DNA, even at gp44:DNA ratios of 100:1, while gp44-CTD-His had the same effect as the full-length gp44, with complete disappearance of the DNA band at high gp44 concentrations (Figure 4B). This suggested that the DNA binding activity resides in the CTD, as expected from the sequence analysis.

**Figure 5:**
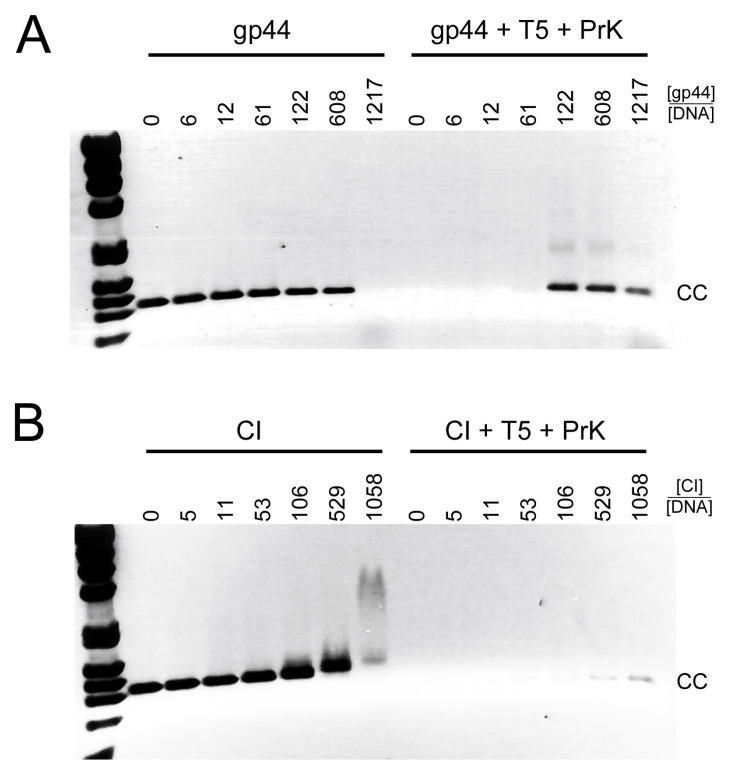
Nuclease protection assay. Agarose gel electrophoresis of 21 nM *cI–cro* (CC) DNA incubated with gp44 (**A**) or CI (**B**) without (left lanes) and with (right lanes) subsequent T5 exonuclease and proteinase K treatment. The -fold molar excess of protein to DNA is shown above the gel. The position of the *cI–cro* DNA is indicated (CC).

We then repeated this experiment with a larger DNA 1136 base pair fragment that included the complete *cI* and *cro* genes as well as the IGR (*cI-cro* DNA). After incubation with CI or gp44, the mixture was separated on a 0.7% agarose gel. The same pattern was observed: CI caused a concentration-dependent shift of the DNA, whereas gp44 caused the DNA band to disappear at high concentration (Figure 5A,B). The amount of shift observed was less in the agarose gel because of the lower resolution, and the protein ratio needed was higher, due to the more stringent conditions (higher salt and EDTA). The disappearance of the gp44 band was accompanied by a corresponding appearance of DNA in the well of the gel (Figure S3).

We interpreted the disappearance of the DNA bands and appearance in the well in the presence of gp44 as caused either by the formation of gp44–DNA supercomplexes that were too large to enter the gel or by neutralization of charges between the positively charged gp44 and the negatively charged DNA. The fact that the gp44-induced shift only occurred at high protein:DNA ratios suggested that the binding was not highly sequence-specific. It is possible that the conditions of the experiment (in the gel) were too harsh for any low-affinity gp44–DNA interactions to be observed. We also considered whether the disappearance of the DNA was caused by degradation by a nuclease contaminant, or because gp44 itself had nuclease activity, but subsequent experiments showed this not to be the case (see below).

### 3.6. Gp44 Protects the Ends of Linear DNA from Degradation

The previous experiments suggested that gp44 is a DNA binding protein. To test whether gp44 could protect DNA from degradation by nucleases, we incubated varying concentrations of gp44 or CI with the *cI–cro* DNA for 15 min, followed by nuclease treatment with either T5 exonuclease or DNase I. T5 exonuclease cleaves only from exposed 5′ ends, leaving the DNA intact if the ends are protected, while DNase I is a non-specific endonuclease that will cleave any unprotected DNA. After 30 min of nuclease incubation at 37 °C, EDTA was added to inactivate the nuclease. The amount of exonuclease, the reaction temperature, and salt concentration were optimized prior to the experiment to ensure that the nuclease would completely cleave unprotected DNA under these conditions.

**Figure 6:**
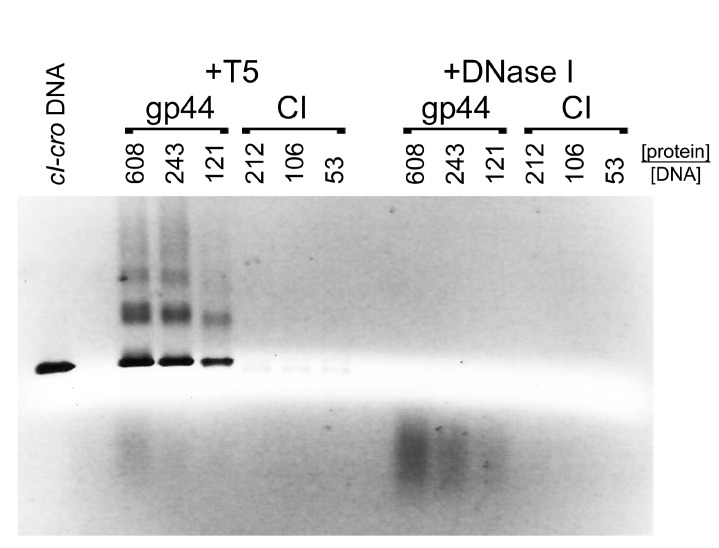
Nuclease protection assay. *cI–cro* DNA was incubated with varying concentrations of gp44 or CI and incubated with either T5 exonuclease or DNase I (endonuclease), followed by proteinase K treatment, showing that gp44, but not CI, protects the DNA against T5 exonuclease, but that neither protein protects the DNA against DNAse I. The -fold molar excess of protein to DNA is shown above the gel.

After incubation of *cI–cro* DNA with gp44 followed by T5 exonuclease treatment, the DNA remained in the well of the agarose gel (Figure S3), suggesting that the large gp44–DNA complexes remained. In order to allow the protected DNA to be analyzed, we added a proteinase K treatment step prior to electrophoresis, which would degrade any proteins and shift the DNA back to its original position in the gel. This experiment showed that incubation with gp44 protected the *cI–cro* DNA from T5 exonuclease digestion in a gp44 concentration-dependent manner, resulting in the reappearance of the *cI–cro* DNA band in the original position (Figure S3, Figure 5A, and Figure 6). In contrast, incubation with comparable amounts of CI did not protect the DNA from T5 exonuclease digestion (Figure 5B and Figure 6). In the presence of DNase I, no DNA was recovered with either gp44 or CI at the same concentrations, showing that neither protein protects the complete DNA from endonuclease digestion (Figure 6). (Although CI is expected to protect a small piece of DNA of about 10–20 nucleotides—60 if it protects both sites simultaneously—this fragment would be too small to show up on the agarose gels.)

To check whether this gp44-induced DNA protection exhibited any specificity for the IGR or *cI–cro* DNA, we repeated the experiment with a 1018 base pair DNA derived from the FibU tail fiber gene *ORF68* (TF DNA). The same behavior was observed as with the *cI–cro* DNA: gp44 protected TF DNA from degradation by T5 exonuclease in a concentration-dependent manner, while CI did not (Figure S4). These results show that gp44, but not CI, binds to the ends of DNA, protecting it from exonuclease digestion, with no sequence preference.

### 3.7. Gp44 Has a Preference for Blunt-Ended DNA

The results above show that gp44 is a DNA binding protein with a preference for the ends of linear DNA. We wanted to know if there was a difference in preference for blunt-ended DNA versus DNA with single-stranded overhangs (sticky ends). To test this, we took full-length 80α phage DNA (43.8 kb) and incubated it with gp44, followed by treatment with either a restriction enzyme that generates sticky ends (EcoRI; 19 cut sites) or one that generates blunt ends (EcoRV; 17 cut sites).

**Figure 7:**
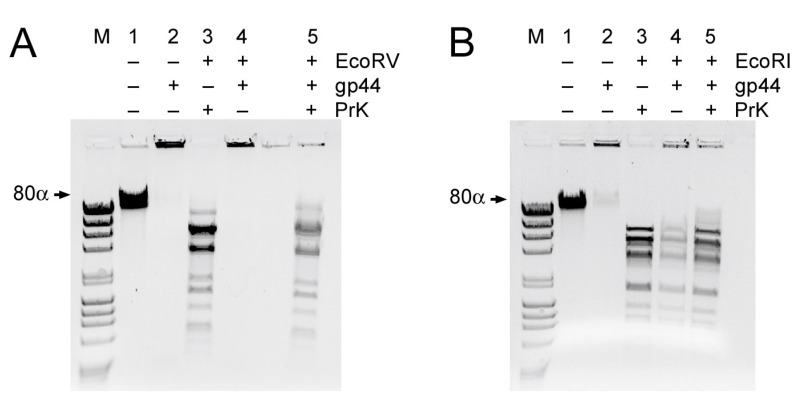
Gp44 preference for blunt-ended DNA. (**A**) 80α genomic DNA (lane 1), incubated with gp44 (lane 2), treated with EcoRV (blunt ends) and incubated with proteinase K (lane 3) or gp44 (lane 4), or treated with gp44 followed by proteinase K (lane 5). (There is a blank lane between lanes 4 and 5.) (**B**) 80α genomic DNA (lane 1), incubated with gp44 (lane 2), treated with EcoRI (sticky ends) and incubated with proteinase K (lane 3) or gp44 (lane 4), or treated with gp44 followed by proteinase K (lane 5).

Uncleaved phage DNA incubated with gp44 under these conditions forms complexes that do not move into the gel (Figure 7), consistent with what we observed before with the *cI–cro* and TF DNA. Cleavage of the DNA with EcoRV (blunt ends) or EcoRI (sticky ends) leads to the formation of a characteristic pattern of bands in the agarose gels (Figure 7A,B). When the DNA was incubated with gp44 prior to EcoRV cleavage, the pattern of bands disappeared and DNA appeared in the well, consistent with the formation of large protein–DNA complexes. Treatment with proteinase K brought most of the DNA back into the gel (Figure 7A). In contrast, when the DNA was treated with EcoRI, most of the DNA ran normally, forming the characteristic pattern of bands in the gel, even in the absence of proteinase K treatment (Figure 7B), indicating that gp44 did not form large complexes with the EcoRI-cleaved DNA. These data show that gp44 binds DNA with a preference for blunt ends over sticky ends.

## 4. Discussion

We previously demonstrated that gp44 is an ejection protein that is required for efficient lytic growth of 80α [11]. Here, we have shown that gp44 is a dimeric protein with affinity for blunt-ended DNA that can protect the DNA from degradation by exonucleases. This suggests that the role of gp44 in the 80α life cycle may be to ensure survival of the genomic DNA after injection and to facilitate its circularization, an essential first step for both lytic growth and establishment of lysogeny.

Gp44-like proteins are common, but specific to headful packaging phages, which constitute the majority of known phages of the Firmicutes [11,19]. Headful packaging phages such as 80α package linear DNA with about 5–10% sequence redundancy. Upon injection, these redundant sequences are used in a recombination reaction to form circular DNA, which is the substrate for replication, and an intermediate in the integration reaction during establishment of lysogeny [17,18]. The phage genome is in a sensitive state immediately after injection, with only a single copy present and presumably various anti-phage defense mechanisms launched to destroy it. Although a certain tolerance of exonuclease digestion is built in due to the sequence redundancy in the genome, a failure to circularize and replicate will cause the DNA to disappear as the cell divides. Once the genome has circularized and started replicating, however, it is more difficult for the host to fight the attack, especially as the phage encodes various functions to suppress normal cell functions [28].

**Figure 8:**
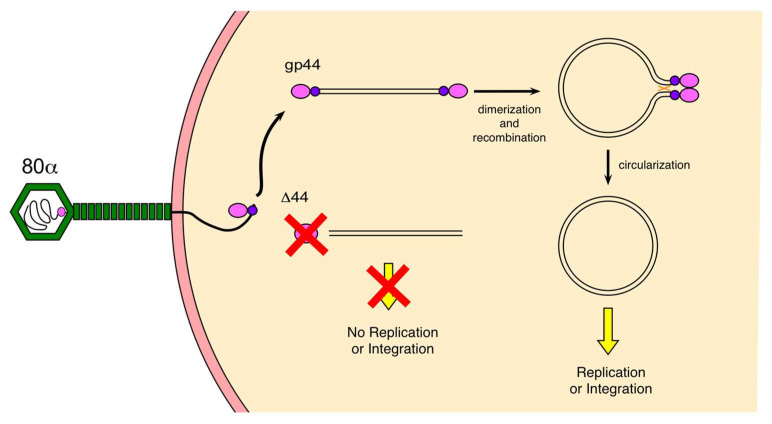
Model for gp44 activity. Gp44 is probably bound to the ends of the genomic DNA inside the phage capsid or binds soon after injection into the host. Dimerization of gp44 leads to loop formation that facilitates recombination and circularization. In the absence of gp44 (80α Δ44), circularization is inefficient, leading to a failure to replicate, and consequently low burst size.

Our data suggest that the role of gp44 is to bind to the ends of the linear genomic DNA after injection, protect it against degradation by host exonucleases, and facilitate its circularization (Figure 8). Headful packaging results in blunt ends and unique end sequences in each virion (albeit with a preference for the *pac* site), consistent with the substrate specificity that we have observed. Dimerization of gp44 bound to the ends of the genome might then lead to the formation of a looped complex that would bring the terminally redundant ends of the genome into proximity, facilitating recombination and circularization by a (host or phage-encoded) recombinase (Figure 8). Gp44 might also assist in the recruitment of the recombinase to the looped complex, although we have no direct evidence for that. Evidently, gp44 is not absolutely required, since some replication occurs even in an 80α Δ44 mutant; however, the burst size is ≈100-fold reduced in the absence of gp44 [11].

It is not known what the recombinase for the circularization reaction is. It could be facilitated by host recombinases, such as RecA; however, most phages also encode their own recombinases that serve a variety of functions [29]. Phage 80α encodes a Sak recombinase (gp16) that has been shown to be essential for lytic growth, both after infection and upon induction of a lysogen [30]. If Sak is required for genome circularization, there is likely to be a time lag before Sak expression is sufficient. Gp44 itself is encoded in the late operon, for which the expression delay would be even longer, which explains why the protein is carried within the capsid itself. We previously calculated that each 80α virion contains about two copies of gp44 [11], suggesting either that one copy is bound to each end of the genome inside the capsid, or that one gp44 dimer is bound to one of the ends. The fact that 80α Δ44 virions carrying gp44 in their virions show higher complementation on a gp44-expressing strain than virions lacking gp44 is consistent with this interpretation [11]. The much greater amount of gp44 needed for DNA protection in vitro than in vivo could be due differences in the in vitro and in vivo milieu, especially considering that the recombinant gp44 protein was produced in *E. coli* and not in its native *S. aureus* host and may not be fully active.

In contrast, the packaged genomes of *cos*-site phages, such as *S. aureus* phage Φ12 or coliphage λ, have sticky ends that do not need a recombination reaction, only ligation, to circularize. Gp44-like proteins have so far not been observed in *cos*-site phages. Perhaps these phages are not as susceptible to exonuclease digestion, or have other mechanisms to protect their DNA after injection, since circularization could happen immediately without the need for recruitment of a recombinase.

We previously observed that lytic growth recovered even after 4 hours of incubation with 80α Δ44 if mitomycin C was added, which originally led us to believe that gp44 was involved in the lytic–lysogenic switch. Clearly, the DNA is able to survive for at least this long without getting degraded, even in the absence of gp44, keeping in mind that the lab strain RN4220 was used and not a “wild-type” *S. aureus* strain that might have more active nucleases. If, as the experiments described here suggest, gp44 is only involved in protection and circularization of the DNA, why would addition of mitomycin C lead to efficient progression to the lytic cycle [11]? While we still do not have a complete answer to this question, it most likely has to do with the SOS response itself, which is activated as a result of DNA damage and activates numerous DNA repair enzymes, including recombinases. It is possible that this increased DNA repair activity will also attempt to repair the linear, unprotected phage DNA.

Our model explains why plasmid transduction is not affected by the lack of gp44. Since plasmids are generally much smaller than the phage genome, they are packaged as linear concatemers into phage capsids [31]. The high sequence redundancy in these concatemers provides a great degree of tolerance of exonuclease cleavage and they supply ideal substrates for recombination. The lack of effect on SaPI transduction in the absence of gp44 was initially harder to explain. SaPIs such as SaPI1 cause the formation of capsids with 1/3 the volume of the phage capsids and should therefore be perfectly matched to the smaller SaPI genome, with similar sequence redundancy to the phage [13,32]. One would expect these genomes to be equally deficient in circularization to the phage genomes, and that SaPIs would display a comparable reduction in transduction titers when mobilized by a gp44-deficient phage. However, results from other groups have suggested that due to the insertion of a tetracycline resistance gene in the SaPI1 genome (SaPI1 *tst::tetM*) used in most experimental studies on SaPI1, the DNA has become too large to be packaged into small capsids [33]. Therefore, only large capsids, containing 2–3 copies of the SaPI *tst::tetM* genome, would be viable. About ≈1–5% of the capsids formed during a typical SaPI1 induction are large, yielding a 20–100-fold reduced transducing titer, but sufficient to produce titers of 10^7^–10^8^ CFU/mL. Due to the added redundancy of these genomes, they would be more tolerant to cleavage and better substrates for recombination, even in the absence of gp44.

Gp44-like proteins appear to be specific to phages of the Firmicutes, which could be related to the presence or absence of specific recombinases or nucleases in these species [11,19]. It is also possible that gp44-like proteins, which are highly divergent, may serve multiple functions. The activity of gp44 is reminiscent of *E. coli* bacteriophage T4 protein gp2, which is a minor structural protein that protects the ends of the genome against degradation by a host exonuclease, and is essential for T4 viability [34]. Interestingly, gp2 is dispensable in a T4 gp23 mutant that makes large heads, presumably due to the added sequence redundancy conferred by packaging multiple copies of the genome, similar to the effect that we have observed with plasmids and SaPIs, described above. Our results suggest that phage ejection proteins involved in genome protection and circularization may be widespread, and the presence of such proteins across a broad range of bacteriophages suggests that these functions are essential for their survival.

## Figures and Tables

**Table 1:** Bacterial strains and plasmids used in this study.

**Strain**	**Genotype/Description**		**Reference**
RN4220	Phage-cured derivative of NCTC8235, modified to accept foreign DNA		[25]
Sa178RI	RN4220 with a chromosomal copy of T7 *pol* gene under *P*_lac_ control (IPTG inducible)		[26]
RN10616	RN4220 (80α)		[14]
ST64	RN4220 (80α Δ44)		[11]
KM12	Sa178RI pKM7		[11]
KM44	Sa178RI pKM13		This study
KM45	Sa178RI pKM14		This study
KM57	Sa178RI pKM28		This study
KM55	Sa178RI pKM24		This study
KM59	Sa178RI pKM29		This study
KM54	Sa178RI pKM27		This study
**Plasmid**	**Derived from**	**Description**	**Source/Reference**
*Escherichia coli* plasmids:			
pET21a	–	*E. coli* expression vector, ampicillin resistance, T7pol promoter	Invitrogen
pKM1	pET21a	Expressing full-length gp44 with C-terminal His6 tag (gp44–His)	This study
pKM9	pET21a	Expressing Gp44-NTD-His (gp44 residues 1–271)	This study
pKM10	pET21a	Expressing Gp44-CTD-His (residues 272–331)	This study
pE-SUMO	pET21a	For expression of proteins with N-terminal His-SUMO tag	LifeSensors
pKM23	pE-SUMO	Expressing His-SUMO-gp44	This study
pKM22	pE-SUMO	Expressing His-SUMO-CI	This study
*Staphylococcus aureus* plasmids:			
pG164	–	Shuttle vector for *S. aureus* expression, T7pol promoter	[26]
pKM7	pG164	Full-length gp44	[11]
pKM13	pG164	gp44-NTD (1–271)	This study
pKM14	pG164	gp44-CTD (272–331)	This study
pKM28	pG164	Gp44-NTD-Long (1–292)	This study
pKM24	pG164	Gp44-NTD-Long+ (1–323)	This study
pKM29	pG164	Gp44-CTD-Long (33–331)	This study
pKM27	pG164	Gp44-CTD-Long+ (8–331)	This study

**Table 2:** Complementation analysis.

Infecting Phage	Recipient Strain ^¶^	Complementing Protein	Residues	IPTG	Phage Titer (PFU/mL)
WT	KM12	gp44 Full Length	1–331	–	10^10^
Δ44	KM12	gp44 Full Length	1–331	–	N.D. ^§^
Δ44	KM12	gp44 Full Length	1–331	+	10^8^
Δ44	KM44	gp44-NTD	1–271	+	N.D.
Δ44	KM45	gp44-CTD	272–331	+	N.D.
Δ44	KM57	gp44-NTD-Long	1–292	+	N.D.
Δ44	KM59	gp44-CTD-Long	33–331	+	N.D.
Δ44	KM55	gp44-NTD-Long+	1–323	+	10^8^
Δ44	KM54	gp44-CTD-Long+	8–331	+	10^8^

^¶^ See Table 1. ^§^ N.D. = not detected; no plaques were observed upon plating undiluted lysate.

## Supplementary Materials

The following are available online at https://www.mdpi.com/1999-4915/12/5/563/s1, Figure S1: Ribbon diagrams of the gp44 structure predictions, Figure S2: Mass spectrometry analysis, Figure S3: Effect of proteinase K treatment, Figure S4: Protection of non-specific DNA.
