# Supplementary material for: The gp44 Ejection Protein of Staphylococcus aureus Bacteriophage 80α Binds to the Ends of the Genome and Protects It from Degradation
Source: Viruses. 2020 May 20;12(5):563. doi: 10.3390/v12050563 (PMC7290940; doi:10.3390/v12050563)

Supplementary figures

Figure S1. Ribbon diagrams of the gp44 structure predictions. Predictions from I-TASSER (A), Phyre2 (B), IntFOLD5 (C) and RaptorX (D), rainbow colored from the N-terminus (blue) to the C-terminus (red). The reliability measure given by the program for each prediction is listed.

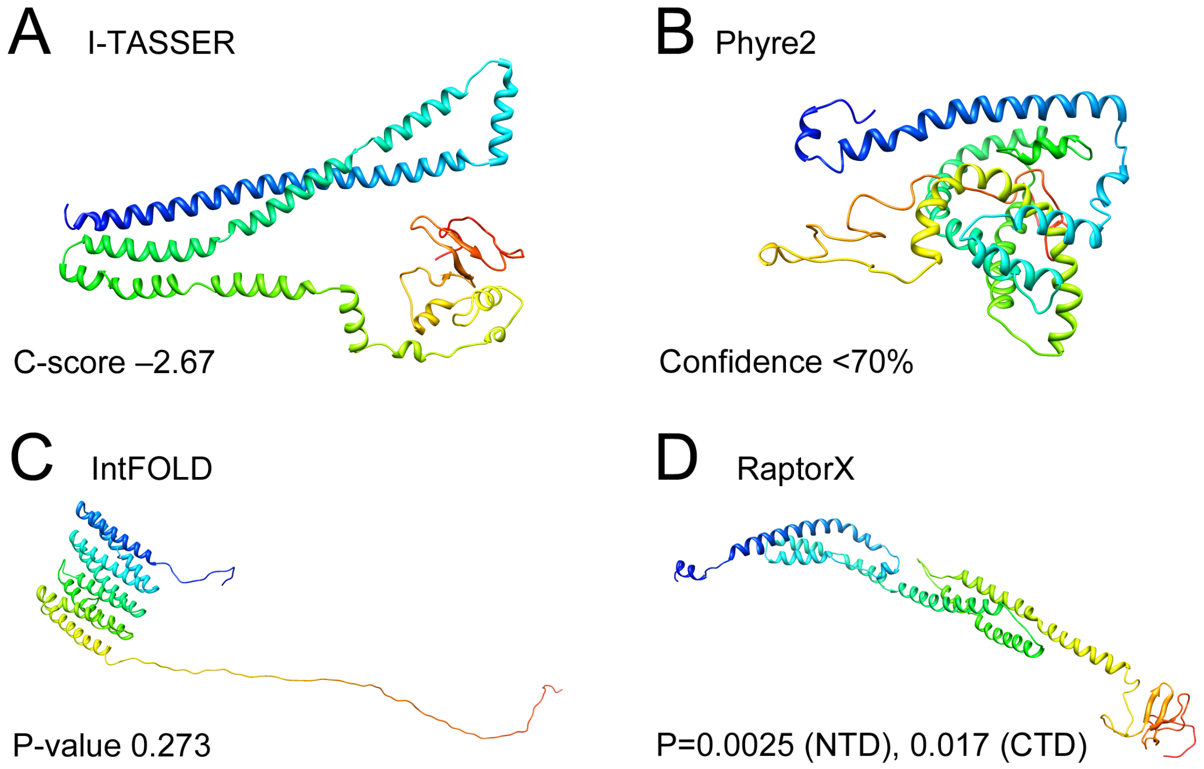

Figure S2. Mass spectrometry analysis. (A-C) LC-MS analysis of the high molecular weight band corresponding to full-length gp44 (A), the intermediate molecular weight band corresponding to gp44-NTD (B) and the low molecular weight band corresponding to gp44-CTD (C), extracted from SDS-PAGE gel of the SEC-purified material (Fig. 2A). The peptides identified in each band are highlighted in yellow over the gp44 sequence. (Methionines and cysteines are shown in green.) (D) Histogram of the number of measured spectra corresponding to the gp44-NTD (blue) and gp44-CTD (orange) in the three bands.

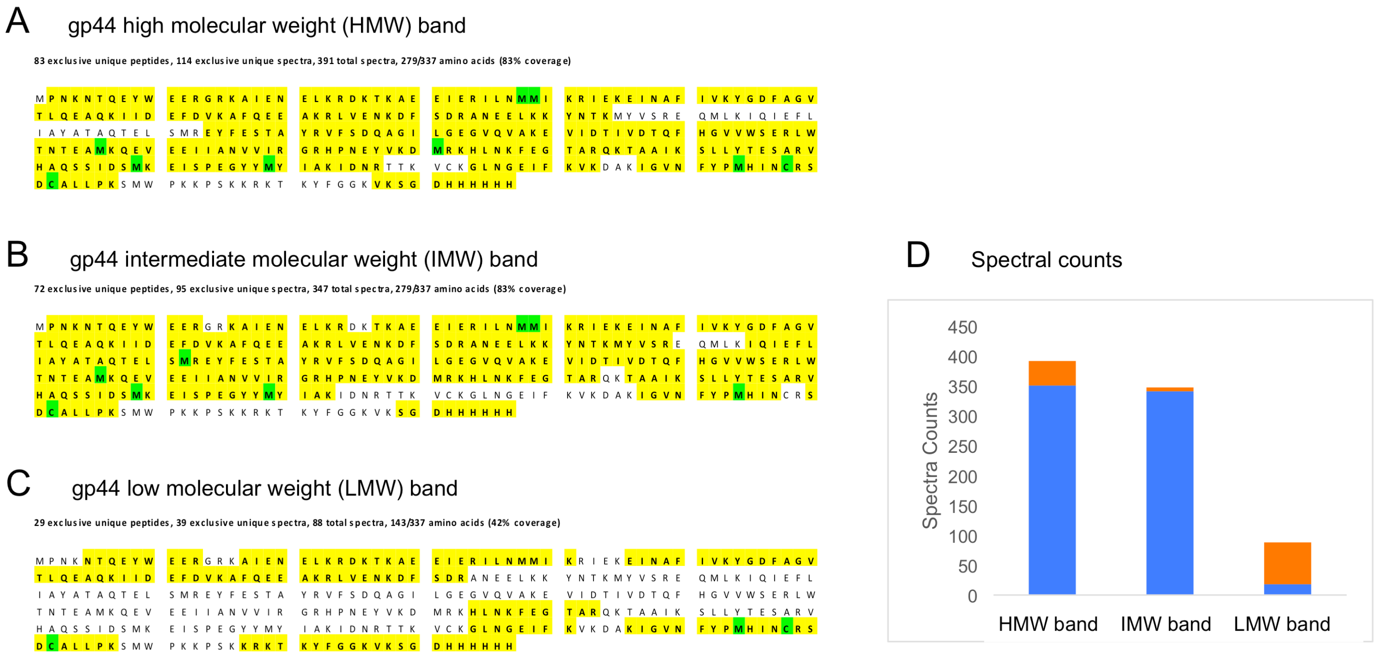

Figure S3. Effect of proteinase K treatment. Agarose gel electrophoresis of 25 nM *cI-cro* DNA incubated with varying concentrations of gp44 followed by T5 exonuclease treatment with (left) and without (right) proteinase K (PrK) treatment. The -fold molar excess of protein to DNA is indicated above the gel. The lane labeled, DNA has no gp44 and no exonuclease; lane 0 has T5 exonuclease, but no gp44. The arrows indicate the position of the *cI-cro* DNA band (CC) and the DNA-gp44 complexes that did not enter the gel (*).

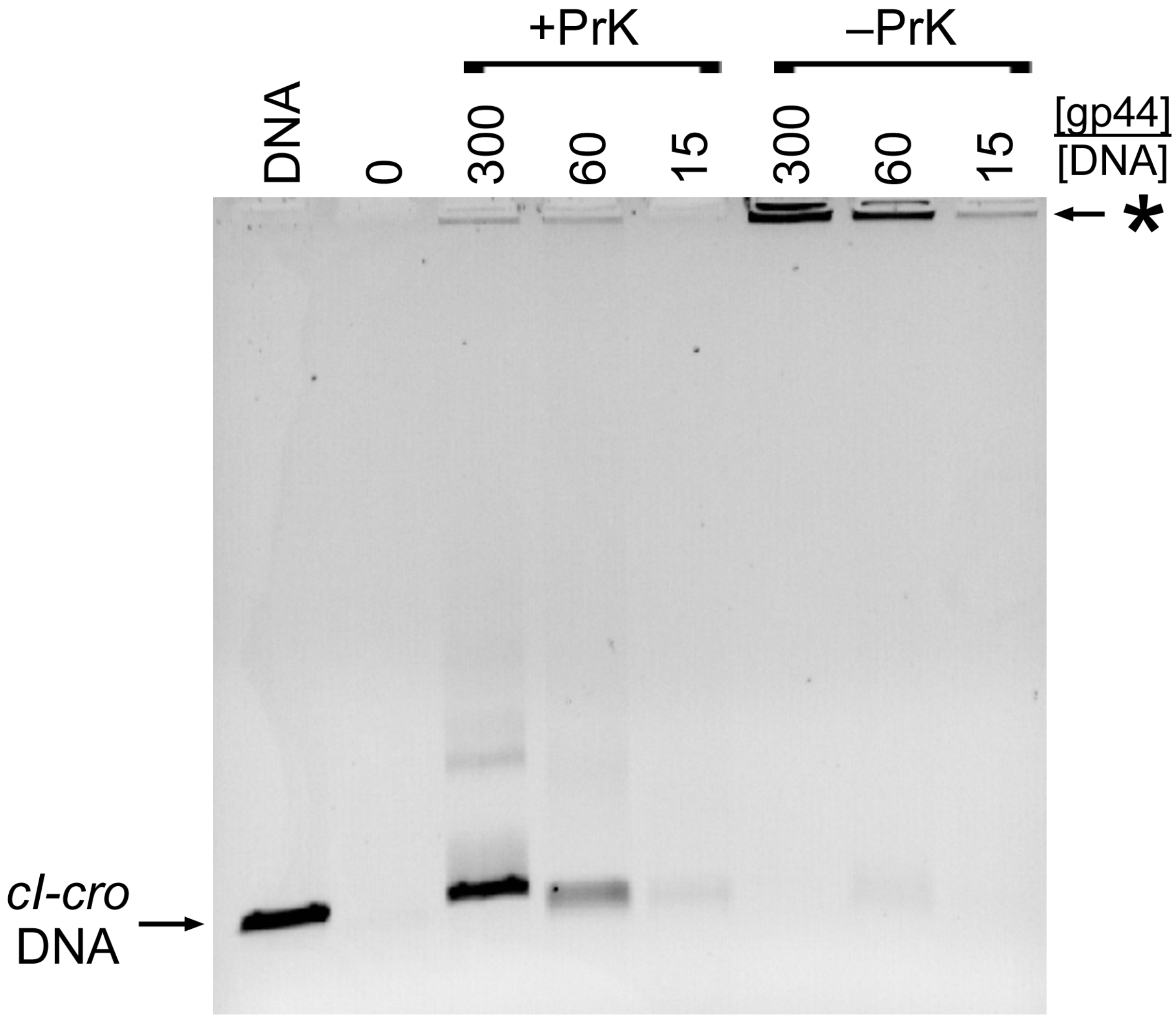

Figure S4. Protection of non-specific DNA. Agarose gel electrophoresis of 25 nM tail fiber (TF) DNA incubated with varying concentrations of gp44 or CI, after incubation with T5 exonuclease, followed by proteinase K treatment, showing that gp44, but not CI, protects TF DNA against T5 degradation. The -fold molar excess of protein to DNA is indicated above the gel. The lane labeled DNA has no gp44 or CI and no T5 exonuclease; lane 0 has T5 exonuclease, but no gp44 or CI.

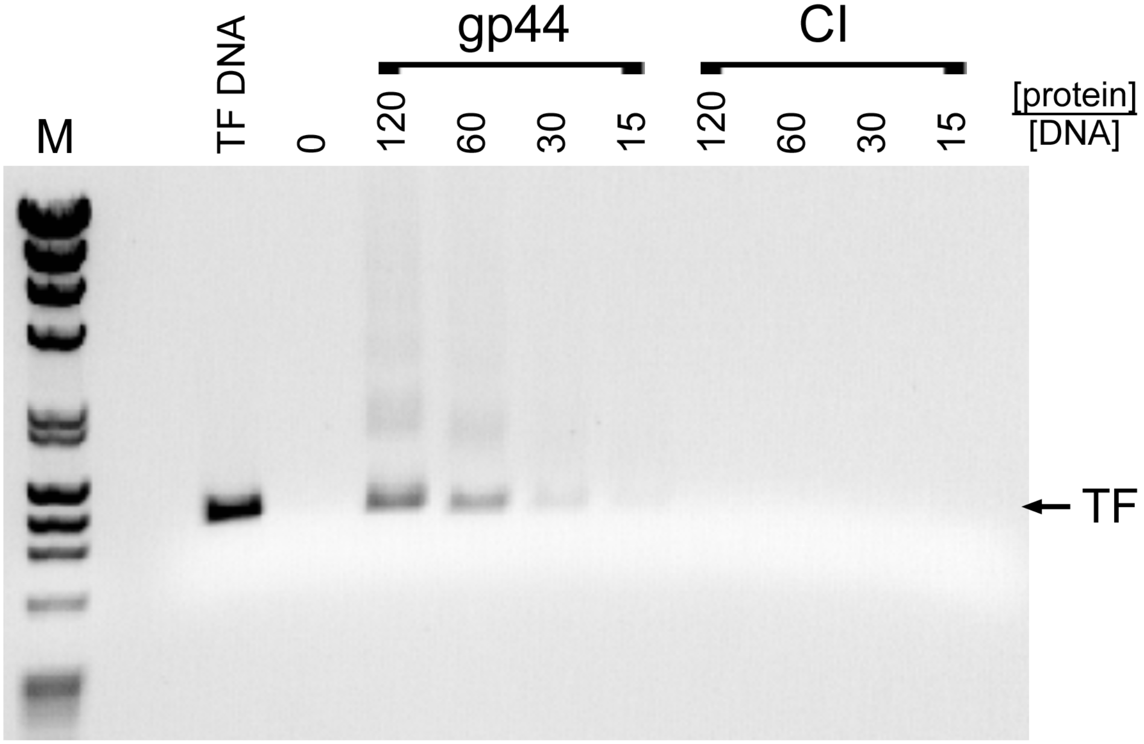

Supplement: Supplementary file 1 [file viruses-12-00563-s001.pdf]
